# Single cells tell it all

**DOI:** 10.7554/eLife.105042

**Published:** 2024-12-23

**Authors:** Margaret Hines, Elias Oxman, Pooja Chauhan, Irene Zohn

**Affiliations:** 1 Center for Genetic Medicine Research at the Children's National Hospital Washington United States

**Keywords:** brain development, scRNAseq, cranial neural plate, diffusion component mapping, single-cell atlas, Mouse

## Abstract

A new single-cell atlas of gene expression provides insights into the patterning of the neural plate of mice.

**Related research article** Brooks ER, Moorman AR, Bhattacharya B, Prudhomme I, Land M, Alcorn HL, Sharma R, Pe’er D, Zallen JA. 2024. A single-cell atlas of spatial and temporal gene expression in the mouse cranial neural plate. *eLife* **13**:RP102819. doi: 10.7554/eLife.102819.

The location of a point in a two-dimensional space can be defined by Cartesian coordinates that indicate its position relative to horizontal and vertical axes. This system was introduced in the 17^th^ century by René Descartes. While it helps find your place on a map, it also captures the journey of cells during embryonic development, particularly in the nervous system. There, gradients of chemical signals known as morphogens form the axes that shape the embryo along anterior-posterior, dorsal-ventral, and mediolateral planes (which run from head to tail, back to belly, and side-to-side). The position of a cell within these gradients will determine the set of genes it expresses and, therefore, its developmental fate and cell identity.

This phenomenon has been best studied in the spinal cord, where cells located ventrally differentiate into motor neurons due to being close to the source of a powerful morphogen called SHH (Tanabe et al., 1995). Under the influence of multiple other morphogens — most notably retinoic acid – different motor neuron types then arise and adopt a precise arrangement along the anterior-posterior spinal cord according to the body regions onto which they project (Stifani, 2014; Liu et al., 2001).

Less is known, however, about how intersecting morphogen gradients such as SHH, WNT, BMP, FGF and retinoic acid contribute to the patterning of the cranial neural plate during early neural development (Kicheva and Briscoe, 2023). The neural plate is a flat sheet of cells that folds onto itself to form the neural tube that will ultimately develop into the brain and spinal cord (Smith and Schoenwolf, 1997). In mice, a key developmental window occurs between 7.5 and 9 days after fertilization (E7.5-E9.0), where the cranial neural plate transforms into a tube while establishing spatial patterns influenced by morphogen gradients (Jacobson and Tam, 1982).

Previous studies into the patterning of neural cells have relied on low-throughput methods to painstakingly examine the expression of genes one at a time (Kicheva and Briscoe, 2023). While these approaches bring precise insights, their stepwise nature limits the pace of discovery. Enter single-cell RNA sequencing (scRNAseq for short), a revolutionary technique that allows gene expression to be simultaneously quantified in tens of thousands of individual cells (Tam and Ho, 2020). Combined with computational approaches, it has allowed researchers to track the trajectories of cells in space and time as they differentiate within a developing embryo. Now, in eLife, Jennifer Zallen and colleagues – including Eric Brooks as first author – report the establishment of a high-resolution single-cell atlas of gene expression during the initial phase of cranial neural plate patterning in mice (Brooks et al., 2024).

The team (based at Sloan Kettering Institute and North Carolina State University) performed scRNAseq on cells from the heads of 30 mouse embryos at six different time points between E7.5 and E9.0. High-quality data were obtained from nearly 40,000 cells, amongst which around 17,500 belonged to the neural plate. To better understand spatial and temporal changes in gene expression amongst these neural cells, Brooks et al. first divided them into four spatial clusters along the anterior-posterior axis based on the expression of a few key region-specific markers. Each cluster was then analyzed using diffusion component mapping, a bioinformatic technique that helps identify and visualize patterns by ordering groups of cells with similar gene expressions. The dominant trend in each cluster identified cells undergoing time-dependent changes in gene expression; this is consistent with the fact that these cells are differentiating from pluripotent stem cells into pro-neurogenic progenitors between E7.5 and E9.0 (Figure 1A).

**Figure 1.**
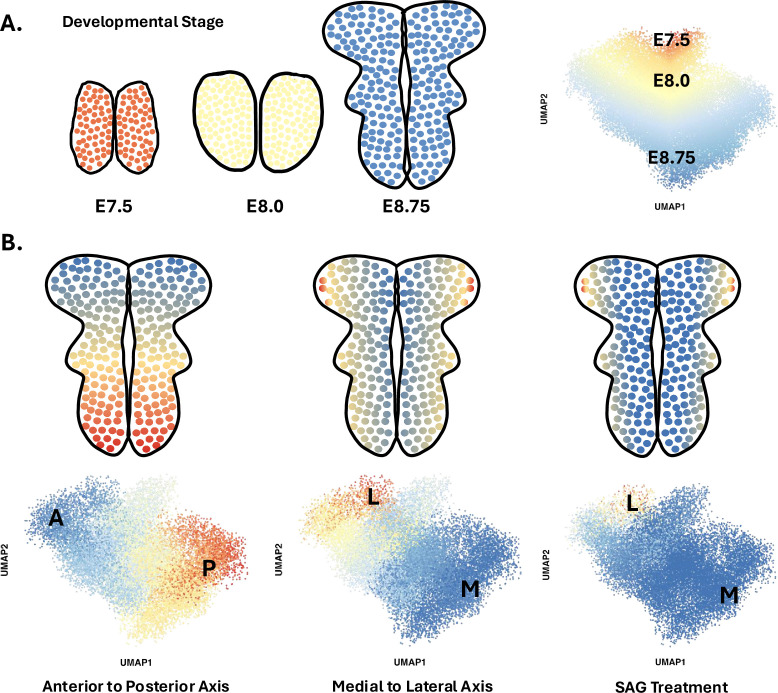
Diffusion component mapping of a robust single-cell RNA sequencing dataset helps to better understand the spatio-temporal dynamics that shape the neural plate of mice during early development. (**A**) Between 7.5 and 9 days after fertilization (stages E7.5 to E9.0), cells in the mouse neural plate (in red, yellow, and blue) undergo differentiation and spatial patterning that results in the formation of the neural tube, a structure that will become the brain and spinal cord. Applying a statistical approach known as diffusion component mapping on a dataset obtained via single-cell RNA sequencing shows that the top diffusion component ordered cells along developmental time (E7.5–9.0) – that is, the main pattern within the data indicates that cell identity shifts over time. This reflects the fact that cells in the neural plate are undergoing differentiation during this period. (**B**) Similar analyses conducted on pooled E8.5–9.0 cells showed that cell identity was strongly spatially patterned at these stages, in particular along the antero-posterior axis (left), but also along the medial to lateral axis (middle). Finally, this approach allowed Brooks et al. to show how a compound known as SAG, which disrupts SHH signalling, can expand ventral cell fates (right).

Next, Brooks et al. focused on how pro-neurogenic progenitors became spatially organized. Diffusion component mapping was again conducted on a subset of the data that pooled neural cells from E8.5–9.0 embryos. A strong spatial signature emerged, with groups of cells being organized along the anterior-posterior axis based on their gene expression signature; the next strongest signal was detected along the mediolateral axis (Figure 1B). By combining this analysis with another computational approach known as HotSpot, the team was able to identify genes whose expression pattern was determined by the position of a cell along the anterior-posterior axis (483 genes), the mediolateral axis (253 genes) or in both dimensions (870 genes).

To assess the validity of this approach, the results were compared to existing data from the Mouse Genome Informatics Gene Expression Database. Information was available for about one-third of the genes with a predicted anterior-posterior expression pattern, confirming that these genes were indeed expressed in the anticipated regions (about 13% were also represented in additional, non-predicted regions but likely at levels below the detection limits of scRNAseq). As for the genes predicted to be mediolaterally patterned, information was present for 65 out of 253 genes, 86% of which were validated in the public databases. These findings demonstrate that scRNAseq analyses can correctly identify known genes expressed in anterior-posterior and medial-lateral organized domains. Significantly, the analysis also uncovered many genes that were previously unknown to be patterned in the developing cranial neural plate.

Further analyses allowed Brooks et al. to infer the function of the patterned genes. A quarter of the anterior-posterior patterned genes and about 30% of the mediolateral-patterned genes were transcriptional regulators, with many known to drive anterior-posterior or mediolateral patterning. The analysis also predicted patterned expression of nearly 200 secreted and transmembrane proteins, which may mediate cell-to-cell communication to establish spatial patterning and differentiation. These include two planar polarity genes required for the proper orientation of cells so that the cranial neural tube can close.

Finally, Brooks et al. used this approach to study how the neural plate becomes mispatterned, particularly when embryos are exposed to a compound known as SAG that causes excess SHH signalling and defects in neural tube closure. These experiments revealed changes in the expression of 365 genes that encode both previously known and unknown components of SHH signaling; half of these genes were found to be spatially regulated in the previous dataset (Figure 1B). Increased SHH signalling also altered the expression of genes under the control of other morphogens. Together, these results suggest that disrupting one signalling axis can have broad implications on how the neural plate is organised in multiple planes and, ultimately, on whether the neural tube forms correctly.

Taken together, the findings illustrate that, when paired with a robust scRNAseq dataset, bioinformatic analysis is a powerful approach that can generate a detailed spatiotemporal map of cell types in the developing nervous system, as well as shed light on how new and known genes are involved in tissue patterning. However, much remains to be explored in this dataset; neural cells represented only a fraction of the data analyzed, and detailed analyses are still to be conducted on other lineages, such as those that will give rise to the bones, vessels, and muscles of the head. Going forward, further analysis of the dataset will provide a useful resource for researchers to generate detailed Cartesian maps of these other tissues.
